# Use of mobile technology for reporting the pharmacovigilance of vaccines in Panama

**DOI:** 10.1016/j.pmedr.2025.103056

**Published:** 2025-03-29

**Authors:** Xavier Sáez-Llorens, Tirza De León, Yostin Jesús Añino, Natalia Vega, Laura Prada, Gabriel Rebollón, Rodrigo DeAntonio

**Affiliations:** a Cevaxin, Centro de Vacunación e Investigación, Av. 3a Oeste, David, Chiriquí, Panama–Cevaxin, Centro de Vacunación e Investigación, Avenida Mexico, Calle 33, Calidonia, Panama City, Panama; b Hospital del Niño Dr. José Renán Esquivel, Avenida Balboa, Calle 34, Panama City, Panama - Sistema Nacional de Investigación, Edificio 205, Ciudad del Saber, Clayton, Panama City, Panama; c Hospital Materno Infantil Jose Domingo De Obaldia, Vía Panamericana, San Pablo Viejo, David, Chiriquí, Panama; d Universidad de Panamá, Campus Dr. Octavio Méndez Pereira, El Cangrejo, Panama City, Panama

**Keywords:** Mobile technology, Pharmacovigilance, Vaccines, Mobile applications, Panama

## Abstract

**Objective:**

Monitoring adverse reactions is essential to confirm vaccine safety profiles. Studies using electronic tools for data collection may reach a broader audience, improving data efficiency and integrity, reducing study costs and simplifying data collection compared with nonelectronic methods. This study aimed to validate electronic versus paper diaries for reporting postimmunization reactions in Panama.

**Methods:**

An experimental design was conducted with three groups (children, pregnant women, and older adults). Groups were divided into one subgroup using paper diary and one using electronic diary. Diary assignments were subsequently reversed in children group, which parents completed. Symptoms and reporting frequency were collected in 2020 and 2021. Information reported in paper diaries was entered into an electronic case report form and reconciled. Users' adherence, differences between reported symptom frequency and users' acceptability of diaries were evaluated.

**Results:**

A total of 180 participants were included: 79 children, 21 pregnant women, 80 older adults. Children group showed greater adherence to both diaries. No significant differences were found in response times in the electronic diary between groups. More symptoms were reported in the electronic diary. The experience of using diaries, no matter which one, was similar.

**Conclusions:**

Results indicate young people adapt better to technological tools than older adults, suggesting tools should be adjusted according to the user's age. Furthermore, electronic applications for reporting postimmunization reactions offer suitable pharmacovigilance alternatives, providing real-time information, and requiring fewer staff, leading to improved health outcomes, patient compliance, and data for research and public health analysis, supporting global vaccine development.

## Highlights

• Electronic tools for reporting postimmunization reactions are feasible in Panama.
• Applications for reporting reactions offer suitable pharmacovigilance alternatives.
• Customizing data collection methods to demographic preferences raises effectiveness.
• Innovative electronic strategies will contribute to global vaccine development.

## 1 Introduction

The number of clinical trials in Latin America has increased in recent decades. According to the Clinical Trials Registry Platform of the World Health Organization, over 150,000 studies are registered in the region (World Health Organization, 2024). Although the development of vaccines and drug pharmacovigilance systems has improved in recent years in Latin America, there are still continuous challenges here (Rodriguez Cadena, 2022; Castañeda-Hernández et al., 2019).

Vaccines are cost-effective interventions, but closely monitoring any potential adverse reactions to confirm the vaccine's safety profile is important (Boersma and Postma, 2021; Aherkar et al., 2016). It is necessary to identify the adverse events related to a specific immunization that do not always occur immediately after vaccination; therefore, the surveillance systems used to monitor vaccine safety are decisive in evaluating the risks and ensuring the population's trust in immunization programs (Larson et al., 2018; Rocca and Anjum, 2020).

To be aware of the occurrence of adverse events postimmunization, the surveillance of vaccine safety is a shared responsibility among governments, the pharmaceutical industry, medical care providers, and the subjects receiving the vaccination (Di Pasquale et al., 2016; Buttery and Clothier, 2022). Studies using electronic tools to collect information have the potential to reach a wider population (van Kerkhof et al., 2016; Scheibe et al., 2015) than other methods (e.g., paper and telephone). The use of diaries may also improve data efficiency and integrity, which may lead to a decrease in the costs of clinical trials and high rates of satisfaction (Haase et al., 2017; Svendsen et al., 2018a; World Health Organization, 2016; Svendsen et al., 2018b). According to the Food and Drug Administration's guidelines for “Electronic Source Data in Clinical Investigations” and “Computerized Systems Used in Clinical Trials,” source data should be “Attributable, Legible, Contemporaneous, Original and Accurate. In the Republic of Panama, most studies use a paper diary for the surveillance of adverse events and the reporting of postvaccination reactions. Several sponsors have requested the use of an electronic diary, but the tool has never been validated in the local context.

It is a priority to enhance safety reports after immunization, whether in the clinical trial context or for national vaccination programs, through the implementation of innovative tools that allow for the collection of information in a timely and accurate manner to monitor the safety of vaccines in children, pregnant women, and adults in Panama. The country has one of the most complete immunization programs in the region, with high vaccination coverage for children and adults (Hodel et al., 2024). The Panama vaccination program, which is known for its comprehensive approach, offers its citizens 23 free and safe vaccines that protect against more than 30 preventable diseases. This program is part of the country's Expanded Program on Immunization (EPI), which aims to ensure high vaccination coverage and reduce the incidence of vaccine-preventable diseases (Rombini et al., 2022).

The present study aimed to validate the use of an electronic diary as a source document to record and monitor adverse events and reactions after immunization and compare its performance with that of paper diaries, here given that, if the validation of the electronic tool results in a profitable and effective tool, this might be replicated in the future to improve safety reports for vaccines in the country.

## 2 Materials and methods

### 2.1 Study design

**Fig. 1:**
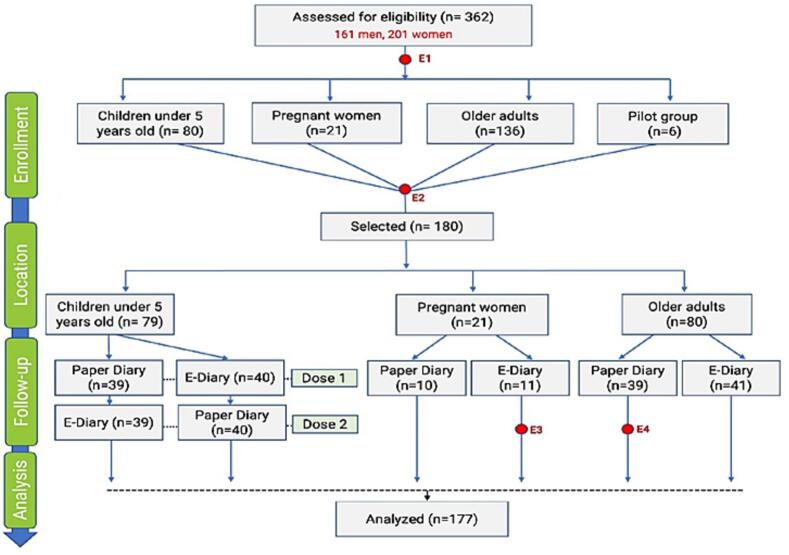
Distribution of participants by number, age group and diary used for recording postimmunization reactions in Panama during 2020–2021.

This was an observational, translational prospective cohort study designed to compare the effectiveness, accuracy, and usability of an electronic diary versus a traditional paper diary across different population groups at two sites in Panama City and one site in David, Republic of Panama. Initially, a pilot study was conducted to validate the paper and electronic diaries. The original design of the diaries was a consensus between the investigational team following a series of discussions through a focus group to verify the accuracy and appropriate language for the target populations. Afterward, three groups were formed as shown in Fig. 1 (children under five years old, pregnant women older than 18 years old, and older adults ≥65 years old) to address three topics: the opportunity to report and access data with both diaries, the adherence of users to each diary, and the acceptability of users.

### 2.2 Participants and data collection period

The participants were eligible to receive vaccinations from the EPI of Panama. The inclusion criteria included the ability to read/write in Spanish and sign an Informed Consent Form (ICF). The participants were provided with either a paper diary or an electronic diary app installed on their smartphones. They were trained on how to complete and report adverse events seven days postvaccination.

Participants were recruited while attending Cevaxin (Center for Vaccines and Clinical Research) to receive their routine immunization. The recruitment period and the data collection period lasted four months for the child group, from 20 August 2020 to 01 December 2020. For the pregnant and older adult groups, the recruitment period lasted five months, from 31 May 2021 to 31 October 2021.

### 2.3 Study procedures

All the groups were divided into two subgroups: one child group was assigned a paper diary, and the other group was assigned an electronic diary. Both diaries were completed by parents/tutors. The assignment of the diaries was reversed, that is, the group that started with a paper diary was then assigned an electronic diary and vice versa. The groups of pregnant women and older adults were divided in a similar way as the child's group; however, these subgroups did not reverse their diary assignments as the child's group did. Focal groups with a duration of approximately an hour were audio-recorded for the qualitative analysis.

In accordance with the EPI recommendations, children in this group were immunized with two doses of the same vaccine. The parents/tutors were given two diaries (in a randomized way, one after the first immunization and the other after the second immunization). The symptoms of the parents/tutors were recorded for seven days postimmunization.

Pregnant women and older adult participants participated for one month. These groups received one immunization and were provided with one diary after immunization. All participants were contacted on 30 days postimmunization to record other possible adverse events.

### 2.4 Sample size calculation

To calculate the sample size, the following formula was used to compare the proportions between the different parameters established in the main objective, with a difference between the paper diary and electronic diary of up to 20 %, via the following formula:

$$n=\frac{N.Z^{2}.p.\left(1-p\right)}{E^{2}.\left(N-1\right)+Z^{2}.p.\left(1-p\right)}$$

where N is the population size, Z is the confidence level value (95 % = 1.96), *p* is the expected proportion of success (*p* = 0.5), and E is the desired margin of error (0.05). Based on these parameters, we estimated a sample size of 80 subjects for each group, including a 10 % loss to follow-up.

### 2.5 Data collection, analysis, and processing

An exact paper and electronic diary were provided to the participants and parents/tutors to complete the report of adverse events following immunization. The information reported in the electronic diary was sent directly to Cevaxin's Patient Follow-up System. The number of symptoms and their frequency of reporting between electronic and paper diaries were collected.

Only the information reported in the paper diaries was entered into the electronic case report form, which is the central database. The medical team clarified the incomplete information with the participants via conciliation. Once the events were conciliated, the medical team performed a one-time acceptability survey consisting of 16 questions with the participants and parents/tutors about their corresponding diaries (See Supplement 1). The survey was evaluated via a Likert scale to analyze the user experience. The parents/tutors of the participants in the child group completed two surveys once they completed each diary. This group received open-ended questions to further analyze their preference between the two diaries. Once the data collection ended, the cost of both diaries was calculated globally and per participant.

### 2.6 Statistical analysis

To analyze the adherence of each group to the follow-up with both electronic and paper diaries, a factorial design was used with analysis of variance tests. The group (children, older adults, and pregnant women) was assigned as factor A, the diary type (electronic and paper) was assigned as factor B, and the response variable was the adherence level, which consisted of a percentage value obtained by dividing the number of days reported by the number of days to be reported. The model was validated by testing the assumptions of normality and homoscedasticity via the Shapiro–Wilk test and Levene's test, respectively.

A Wilcoxon signed-rank test (W test) was used to determine if there were significant differences in the frequency of reported symptoms. This test was selected after validating that the assumptions of normality and homoscedasticity were not met. Similarly, this test was used to assess the acceptability of both diaries among users.

The software used for the analysis was Excel and dynamic tables in the R program for inferential statistical analysis.

### 2.7 Ethics approval and consent to participate

The study was reviewed and approved by the Hospital del Niño Dr. José Renán Esquivel (Doctor José Renán Esquivel Children's Hospital) Committee of Bioethics in Investigation. Prior to survey completion, all pregnant women and older adult participants read, completed, and signed the approved ICF, and written informed consent was obtained. Likewise, written informed consent was obtained from the parents/tutors of each participant under five years old. The signed ICFs were archived in the study file.

## 3 Results

A total of 362 participants were screened, and 180 were included. Fig. 1 shows the allocation of participants to each of the study groups.

### 3.1 Adherence level

**Fig. 2:**
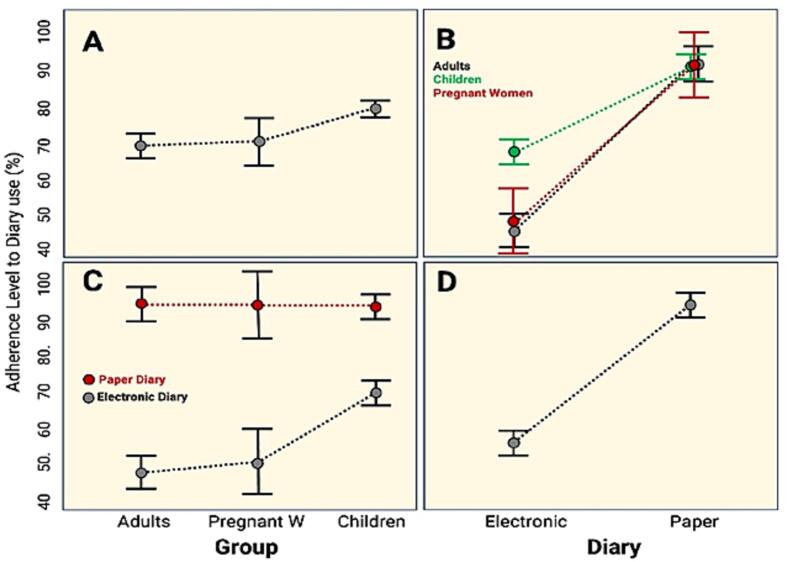
Comparisons of adherence level of participants by group and by diary type used for recording postimmunization reactions in Panama during 2020–2021.

The results revealed significant differences between the groups (*p* value = 0.03), with the children's group having greater adherence than those in the other two groups. Similarly, there were differences in adherence levels between the two diaries (*p* value ≤0.01), with the paper diary having the highest adherence level. Fig. 2 shows the group's adherence to the diaries.

### 3.2 Frequency of reporting symptoms

**Fig. 3:**
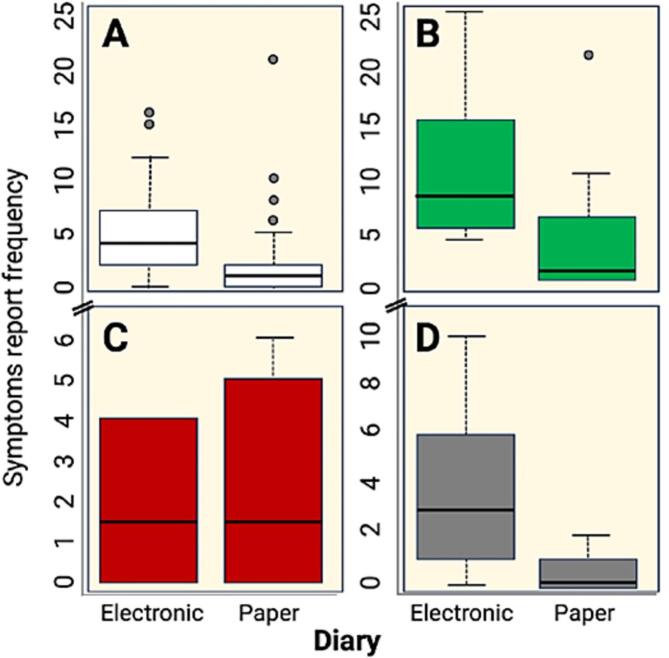
Frequency in reporting symptoms by type of diary, by study group, and by all study groups for postimmunization reactions in Panama during 2020–2021.

Differences in the frequency of symptom reporting (W test = 531.5, *p* value = 0.01) were observed, with the electronic diary showing a greater frequency of reports. In the children (W test = under *p* value = 0.01) and older adults (W test = 88.5, *p* value = 0.01) groups, the frequency of reporting symptoms was greater in the electronic diary. In the pregnant group, no significant differences were observed between the diaries (W test = 19.5, *p* value = 0.77; see Fig. 3).

### 3.3 Acceptability of both diaries by users

Acceptability in all three groups ranged from 91 % to 100 %. The pregnant women in the group with electronic diaries and the older adult group with paper diaries did not have a 100 % acceptability rate. According to the satisfaction survey, there were no differences between aspects related to experience (W test = 366.5, *p* value = 0.39), instructions in both diaries (V = 161.5, *p* value = 0.98), language used (W test = 172.5, *p* value = 0.30), difficulty level (W test = 42.5, *p* value = 0.12), and the clarity to complete them (W test = 42.5, *p* value = 0.12). The participants reported that they would participate in a study if they were asked to complete an electronic or paper diary (W test = 37.5, *p* value = 0.37).

The survey included four items for comparisons between diaries (See supplement 1). For the comparisons, the median was considered because it is the best measure of central tendency to explain the obtained data. The three groups were compared through the reporting of symptoms and frequency of reporting in each of the diaries. According to the survey, the diary that required the longest time to be completed was the paper diary, while the electronic diary was, according to users, more user friendly and convenient, which is why there was a greater frequency of responses that recommended the use of the electronic diary. The full data set is available upon request.

## 4 Discussion

Working in the context of Panama, the present study aimed to validate the use of an electronic diary compared with a paper diary for reporting adverse events and reactions after immunization. This is the first tool used to report symptoms and adverse events in the context of the EPI in the country and the Central American region. Given the lack of studies evaluating such tools in the region, our results hold significance; they provide key insights and data that were previously unavailable, highlighting the need for continued monitoring and research in this area.

Our findings demonstrate that reporting symptoms via an electronic diary is feasible in populations of different age groups. Similarly, a dermatology trial assessed the use of an e-diary to report events and measure adherence and reported high treatment adherence rates (median 98 %) and high e-diary adherence for capturing drug administration and symptoms, with patients rating the e-diary highly for user acceptability (Pan American Health Organization, 2020). Both studies underline the effectiveness and user preference of electronic diaries in different contexts, reinforcing their potential for improving data collection and adherence in clinical settings. Similarly, another study reported that 78 % of patients completed the 12-month study period, and 55 % submitted at least 75 % of the requested surveys, showing strong adherence to the e-diary system and demonstrating that changes in patient reporting outcomes derived from the e-diary over time could predict clinical relapses, hence emphasizing the potential of e-diaries for improved clinical research and patient care in real-world settings (Rijsbergen et al., 2020).

The findings of our study provide evidence of the relative strengths and weaknesses of electronic versus paper data collection methods in diverse populations. The insights can help inform best practices for selecting data collection tools in public health research, particularly in settings where accurate and efficient data collection is essential. In the clinical trials setting, smartphones and data plans are always provided for the participants to ensure compliance with reporting safety information in a timely manner. For post-licensure safety surveillance initiatives, these e-tools facilitate real-time data collection and reporting. This approach can be extended to post-licensure initiatives, where the widespread availability of smartphones and internet connectivity in Panama ensures that the population eligible for vaccination can easily access and use these tools, enhancing participant engagement and compliance (Branch, Statistics on Panama's Digital Situation in, 2024).

Furthermore, our study revealed demographic-specific preferences or barriers, aiding in the customization of data collection strategies for different age groups or populations with varying levels of digital knowledge, which can be compared with the findings of a cross-sectional study that suggested that while many individuals use health apps, a substantial proportion of the population does not and that even among those who use health apps, many stop using them. These data suggest that app developers need to better address consumer concerns (Krebs and Duncan, 2015).

The development of the focal group process allowed the participants to validate the tool's validity. Moreover, all participants received training instructions on completing both diaries, which could result in greater use of the electronic instrument, as reported in other studies using this electronic tool (Haase et al., 2017; World Health Organization, 2016; Golan et al., 2021; Krogh et al., 2016).

The increased effectiveness and preference for electronic diaries among users can significantly influence their future use. According to another study, factors such as reminders, attractive designs, tailored data visualizations, and user characteristics such as smartphone experience and intrinsic motivation to change behavior play decisive roles in the adoption and sustained use of electronic diaries in healthcare (Daniëls et al., 2021). With features such as real-time updates, synchronization across multiple devices, and enhanced safety, electronic diaries offer convenience and functionality that traditional paper diaries cannot. One qualitative study underscores that users appreciate the technical functionality, ease of use, and design features of mobile health apps, which facilitate self-care and encourage persistent use (Tang et al., 2015). As users continue to favor these digital tools, their developers will presumably invest more in improving the user experience, focusing on aspects such as engagement, technical functionality, and data management to meet user expectations and needs. The highest adherence level and preference for reporting symptoms using the electronic diary instead of the paper diary are in line with what has been reported in previous studies.

The acceptability of the electronic diary was similar to that of previous studies that used electronic tools, where the participants reported no significant issues with the use of this type of application and considered them efficient tools (World Health Organization, 2016; Hodel et al., 2024; Krogh et al., 2016; Daniëls et al., 2021). Considering that the electronic diary was well accepted in the present study, it could be used to record symptoms and reactions after immunization in a timely manner.

The study was conducted during the pandemic, which allowed monitoring for contingency plans in public health situations. The opportunity to report and access data could not be compared between the electronic and paper diaries because access to data in real time was not comparable between the two diaries. In addition, the participants were not contacted by telephone to check if they were completing their paper diaries because this was not within the scope of the study.

Regarding the survey related to acceptability and the responses showing no significant differences between one diary and the other, the questions were not designed to compare efficiency between the two diaries but rather were designed separately.

### 4.1 Implications

Owing to their enhanced effectiveness, the preference for electronic diaries over traditional paper diaries could revolutionize their implementation in clinical and public health settings. With features such as automated data entry, real-time tracking, and integration with electronic health records, electronic diaries offer a streamlined approach to patient monitoring and data collection. This can lead to more accurate and timely health interventions, improved patient compliance, and more comprehensive data for research and public health analysis. Furthermore, the ease of use and accessibility of electronic diaries could encourage wider patient participation in health monitoring programs, hence leading to better health outcomes and more efficient healthcare delivery systems (Golan et al., 2021; Anderson et al., 2016; Nieuwlaat et al., 2014; Endalamaw et al., 2024).

### 4.2 Limitations

Because this was a study that did not follow up with the subjects so that they could report during the seven days requested, much of the data were not recorded in either diaries in terms of days to report or the frequency of the type of symptoms.

The measure used to collect the data were affected because, when recording the information obtained from the paper diaries, the word “not applicable” (NA) was not included in some of the symptoms, where it can be inferred that the subject did not mark the symptoms in the diary.

One significant limitation was the COVID-19 pandemic, which raised a challenge primarily because it altered the originally planned timelines in the study protocol, which had been approved before the pandemic started. In addition, the pandemic interfered with the completion of the expected sample size, particularly within the pregnant women group, who were considered a high-risk population during this period. Furthermore, costs escalated because of the need for personal protective equipment and other pandemic-related expenses.

## 5 Conclusion

The present study demonstrated that the use of electronic diaries for reporting adverse events and reactions after immunization is feasible across different age groups in Panama. This represents the first use of such a tool in the context of the EPI in the country and Central American region. Similarly, the study demonstrated demographic-specific preferences and barriers, which can help customize data collection strategies for different age groups and populations with varying levels of digital knowledge. The study validated the usability of electronic diaries over paper diaries for reporting adverse events after immunization and showed that electronic diaries are feasible across different age groups in Panama, hence providing insights into the strengths and weaknesses of both methods and emphasizing a greater preference and adherence to electronic diaries because of their convenience and advanced features.

Electronic diaries can revolutionize pharmacovigilance by enabling real-time, accurate data collection and integration with electronic health records. This leads to faster identification of adverse reactions, improved patient compliance, and more comprehensive data for analysis. The ease of use and accessibility of electronic diaries encourage wider patient participation, ultimately enhancing drug safety and health outcomes. Thus, given its potential to enhance safety and health outcomes, further research into the use of electronic tools in pharmacovigilance is highly recommended.

The results indicate that young people are more familiar with and comfortable with technological tools than older adults are. Electronic tools that report symptoms and adverse events after immunization should be considered promising alternatives to collect reliable diary data in a real-world setting and improve safety reports for vaccines in the Central American region. The acceptability of the electronic diary in different age groups makes it a friendly tool to be used by anyone; however, these tools must be adjusted to the user according to age. The implementation of innovative electronic strategies, even without the need for connection, will allow us to continue contributing to the global development of vaccines.

## CRediT authorship contribution statement

**Xavier Sáez-Llorens:** Writing – review & editing, Writing – original draft, Validation, Supervision, Methodology, Conceptualization. **Tirza De León:** Writing – review & editing, Methodology, Conceptualization. **Yostin Jesús Añino:** Writing – review & editing, Writing – original draft, Validation, Software, Methodology, Investigation, Formal analysis. **Natalia Vega:** Writing – review & editing, Validation, Conceptualization. **Laura Prada:** Writing – review & editing, Validation, Conceptualization. **Gabriel Rebollón:** Writing – review & editing, Visualization, Conceptualization. **Rodrigo DeAntonio:** Writing – review & editing, Writing – original draft, Validation, Supervision, Methodology, Investigation, Conceptualization.

## Funding

This project was supported by grant number FID18–068. The Secretaria Nacional de Ciencia, Tecnología e Innovación de la República de Panamá, SENACYT, (National Secretariat of Science, Technology and Innovation of the Republic of Panama, SENACYT) funded the study as part of the Public Call for Investigation and Research Promotion 2018, Republic of Panama. https://www.senacyt.gob.pa/. Likewise, Cevaxin provided its own resources to conduct this project. XSZ, TDL, YA, NV, LP, GR, and RDA received the grant. The funders had no role in study design, data collection and analysis, the decision to publish, or the preparation of the manuscript. At the time of the study, RDA was part of the SENACYT investigators and received a salary at the time. XSZ receives a salary from SENACYT and is part of their investigators.

## Declaration of competing interest

The authors declare the following financial interests/personal relationships which may be considered as potential competing interests: Xavier Saez-Llorens reports a relationship with Secretaría Nacional de Ciencia, Tecnología e Innovación that includes: consulting or advisory. Rodrigo DeAntonio reports a relationship with Secretaría Nacional de Ciencia, Tecnología e Innovación that includes: consulting or advisory. This project was supported by grant number FID18–068. The Secretaria Nacional de Ciencia, Tecnología e Innovación, SENACYT, funded the study as part of the Public Call for Investigation and Research Promotion 2018, Republic of Panama. https://www.senacyt.gob.pa/. Likewise, Cevaxin provided its own resources to conduct this project. XSL, and RDA received the grant. The funders had no role in study design, data collection and analysis, the decision to publish, or the preparation of the manuscript. At the time of the study, RDA was part of the SENACYT investigators and received a salary at the time. XSL receives a salary from SENACYT and is part of their investigators. If there are other authors, they declare that they have no known competing financial interests or personal relationships that could have appeared to influence the work reported in this paper.

## Data Availability

Data will be made available on request.

## Appendix A Supplementary data

Supplementary material Image 1
