# Supplementary material for: Use of mobile technology for reporting the pharmacovigilance of vaccines in Panama
Source: Prev Med Rep. 2025 Mar 29;53:103056. doi: 10.1016/j.pmedr.2025.103056 (PMC11997413; doi:10.1016/j.pmedr.2025.103056)
Supplement: Supplementary file 1 — Supplementary material [file mmc1.docx]

**Supplement 1. Acceptability survey- electronic and paper diary**

**Items and weights of the satisfaction survey**

**Questions:**

1. Your experience using the paper diary was:
2. The instructions provided in the paper diary were:
3. The language used in the paper diary was:

**Possible responses:**

1. Very unsatisfied/Very unsatisfactory
2. Unsatisfied/Unsatisfactory
3. Moderately satisfied/Unsatisfactory
4. Satisfied/Satisfactory
5. Very satisfied/Satisfactory

**Questions:**

1. Did you have any difficulties responding on the paper diary?
2. Was it easy to complete the paper diary?
3. Do you consider you would participate in a study if they asked you to complete a paper diary?

**Possible responses:**

1. I don’t know
2. No
3. Yes

**Questions:**

1. Your experience using the electronic diary was:
2. The instructions provided in the electronic diary were:
3. The language used in the electronic diary was:

**Possible responses:**

1. Very unsatisfied/Very unsatisfactory
2. Unsatisfied/Unsatisfactory
3. Moderately satisfied/Unsatisfactory
4. Satisfied/Satisfactory
5. Very satisfied/Satisfactory

**Questions:**

1. Did you have any difficulties responding on the paper diary?
2. Was it easy to complete the paper diary?
3. Do you consider you would participate in a study if they asked you to complete an electronic diary?

**Possible responses:**

1. I don’t know
2. No
3. Yes

**Questions:**

1. Which of the two diaries took the longest to respond to?
2. Which of the two diaries did you find more user-friendly?
3. Which of the two diaries do you find more convenient to complete based on your lifestyle?
4. Overall, which of the two diaries would you recommend?

**Possible responses:**

1. There was no difference
2. Electronic Diary
3. Paper Diary
